# Characterization of 20 complete plastomes from the tribe Laureae (Lauraceae) and distribution of small inversions

**DOI:** 10.1371/journal.pone.0224622

**Published:** 2019-11-01

**Authors:** Sangjin Jo, Young-Kee Kim, Se-Hwan Cheon, Qiang Fan, Ki-Joong Kim

**Affiliations:** 1 School of Life Sciences, Korea University, Seoul, Korea; 2 School of Life Sciences, Sun Yat-sen University, Guangzhou, China; Institute for Biological Research, SERBIA

## Abstract

*Lindera* Thunb. (Lauraceae) consists of approximately 100 species, mainly distributed in the temperate and tropical regions of East Asia. In this study, we report 20 new, complete plastome sequences including 17 *Lindera* species and three related species, *Actinodaphne lancifolia*, *Litsea japonica* and *Sassafras tzumu*. The complete plastomes of *Lindera* range from 152,502 bp (*L*. *neesiana*) to 154,314 bp (*L*. *erythrocarpa*) in length. Eleven small inversion (SI) sites are documented among the plastomes. Six of the 11 SI sites are newly reported and they locate in *rpo*B-*trn*C, *psb*C-*trn*S, *pet*A-*psb*J, *rpo*A and *ycf*2 regions. The distribution patterns of SIs are useful for species identification. An average of 83 simple sequence repeats (SSRs) were detected in each plastome. The mono-SSRs accounted for 72.7% of total SSRs, followed by di- (12.4%), tetra- (9.4%), tri- (4.2%), and penta-SSRs (1.3%). Of these SSRs, 64.6% were distributed in an intergenic spacer (IGS) region. In addition, 79.8% of the SSRs are located in a large single copy (LSC) region. In contrast, almost no SSRs are distributed in inverted repeat (IR) regions. The SSR loci are useful to identifying species but the phylogenetic value is low because the majority of them show autapomorphic status or highly homoplastic characteristics. The nucleotide diversity (Pi) values also indicated the conserved nature of the IR region compared to LSC and small single copy (SSC) regions. Five spacer regions with high Pi values, *trn*H-*psb*A, *pet*A-*psb*J and *ndh*F-*rpl*32, *rpl*32-*trn*L and Ψ*ycf*1-*ndh*F, have a potential use for the molecular identification study of *Lindera* and related species. *Lindera* species form a paraphyletic group in the plastome tree because of the inclusion of related genera such as *Actinodaphne*, *Laurus*, *Litsea* and *Neolitsea*. A former member of tribe Laureae, *Sassafras*, forms a clade with the tribe Cinnamomeae. The SIs do not affect the phylogenetic relationship of Laureae. This result indicated that ancient plastome captures may have contribute to the mixed intergeneric relationship of Laureae. Alternatively, the result may indicate that the morphological characters defined the genera of Lauraceae originated for several times.

## Introduction

Lauraceae belong to the order Laurales and include approximately 45 genera and 2,850 species **[1, 2]**. They are widely distributed in temperate and tropical regions and are mainly distributed in Southeast Asia and South America **[3]**. Some of its species are economically important crops that are used as medicine, timber, fruit, and perfume **[3]**. Systematically, Lauraceae form close relationships with Hernandiaceae and Monimiaceae **[4, 5]**. Lauraceae are largely classified into core groups consisting of Laureae, Cinnamomeae, and Perseae, and other basal groups **[6]**.

The tribe Laureae is composed of about 500 species in 10 genera (*Actinodaphne* Nees, *Dodecadenia* Nees, *Iteadaphne* Blume, *Laurus* L., *Lindera* Thunb., *Litosea* Lam., *Neolitsea* (Benth. & Hook.f.) Merr., *Parasassafras* Long, *Sassafras* J.Presl and *Sinosassafras* H.W.Li) and accounts for about 18% of Lauraceae **[3, 7]**. All species in Laureae are dioecious and most of them have umbellate inflorescences wrapped by bracts, and introse anthers **[6]**. Except for *Sassafras* (including uni- and bisexual), all nine genera are unisexual. *Lindera*, a representative genus of Laureae, is evergreen or deciduous trees or shrubs, and about 100 species **[3]**. Although most species are distributed in the temperate and tropical regions of East Asia, some species are distributed in North America too **[8]**. In East Asia, four Korean species, 38 Chinese species, and seven Japanese species are known **[3, 9, 10]**. *Litsea* is evergreen or deciduous trees or shrubs and includes about 200 species. It is mainly distributed in tropical and subtropical Asian regions and also rarely distributed in Australia and America. *Lindera* and *Litsea* are mostly evergreen, but they include some deciduous plants **[3, 8–10]**. In contrast, *Sassafras* is composed of three species, all of which are deciduous **[3, 8–10]**.

Plastid DNA is the most important molecular marker in plant systematics. A few plastid genes were employed to elucidate the phylogenetic relationships of various plant groups until year 2000 **[11]**. With the development of sequencing technology, phylogenetic studies have evolved from using a few gene sequences to complete plastome sequences. Currently, complete plastomes from more than 2,000 plant species are available from NCBI database. In angiosperms, most of the autotrophic plant species have approximately 100–112 unique genes in the plastome **[12]**. The sizes of plastomes usually range from 120 to 180 kb in size **[12]**.

The complete plastomes of Laurales have been reported from two species of Calycanthaceae **[13]** and 44 species of Lauraceae **[14–25]**. However, the sequences of these 35 species are available in the NCBI database (checked on May 7, 2018). In addition, among the 35 published species, the plastome sequences of only 11 genera and 24 species have been fully verified because the plastome sequences of 9 genera and 11 species are unverified and have not been completely annotated.

Except for the parasitic plant *Cassytha*, no large structural variation or peculiarity in gene content has been found on the plastomes of Lauraceae **[20, 21]**. Only minor contractions/expansions of an inverted repeat (IR) region have been reported on the large single copy (LSC)/IR region boundaries of Lauraceae plastomes **[20, 21]**. Phylogenetic studies on Lauraceae using a few chloroplast gene sequences have been actively conducted over the last 20 years **[6, 26–30]**. According to these studies, the *Lindera* spp. formed polyphyletic clades because they are mixed with species from other genera **[26, 27]**. Recent systematic studies using all plastid coding gene sequences yield identical results to studies that used a few gene sequences **[26, 27]**. A main morphological difference between *Lindera* and *Litsea*, which overlap in molecular trees, is two-celled versus four-celled anthers **[26, 27]**. Despite the morphological difference and increased molecular data, the phylogenetic relationship within the Laureae remains unclear.

The large inversion (LI) of plastid genomes is occasionally reported from several plant families, such as Asteraceae **[31]**, Fabaceae **[32, 33]**, Geraniaceae **[34, 35]**, Oleaceae **[36, 37]**, Passifloraceae **[38]**, and Poaceae **[39].** The LIs are often showed the systematic utilities because they occur in a clade of certain groups. Conversely, small inversion (SI)s occur extensively in any studied angiosperm plastome **[31, 40]**. Previously, based on partial sequences, the presence of SIs was reported only in certain regions of plastome. In particular, it has been reported in the *ndh*B intron **[41]**, *trn*H-*psb*A **[42–45]**, *pet*A-*psb*J **[46]**, *rpl*16 intron **[47]** and *trn*L-F regions **[42, 48]**. SIs are not easily found because they are usually located in spacer regions and do not affect the gene order. Therefore, not only have there been few studies on SIs, but these studies have found a limited number of regions of SIs. As complete plastome studies have been developing, the areas where SIs are found are increasing. For examples, Kim and Lee (2005) compared the complete plastomes of four species of Poaceae and reported 16 SI regions **[31]**. Thereafter, SIs have been reported in studies of Araliaceae **[49]**, Arecaceae **[50]**, Lauraceae **[14, 15, 18, 19]**, Lamiaceae **[51]**, and Oleaceae **[37]**.

In this study, we report 20 complete plastome sequences from Lauraceae. Seventeen of them belong to *Lindera* and the other three species are *Actinodaphne lancifolia* (Blume) Meisn., *Litsea japonica* (Thunb.) Juss. and *Sassafras tzumu* (Hemsl.) Hemsl. In addition to our 20 new plastome sequences, all available plastome sequences in the NCBI database were compared and analyzed to investigate the systematic relationships of *Lindera* and closely related taxa. First, the degrees and locations of SIs of these plastomes were identified, and then whether these inversions affect the construction of phylogenetic trees was evaluated. Second, the hotspot regions of plastomes, which are important for interspecific relationships, were evaluated, and simple sequence repeats (SSRs) and dispersed repeat sequences are reported. Third, the phylogenetic relationships of the tribe Laureae using coding, noncoding, and all sequences, are compared. Finally, the evolution patterns of traits such as evergreen and deciduous are discussed.

## Materials and methods

### Plant materials and DNA extraction

The leaves of 17 *Lindera* and three outgroup species used in this study were collected from Korea, China and Japan. All voucher specimens were deposited in the Korea University Herbarium (KUS). Their information is summarized in Table 1. Fresh leaves were collected and ground into powder in liquid nitrogen for Korean materials. Collected leaves are dried in silica gel and transported to lab for Chinese and Japanese materials. Total DNAs were extracted using a plant genomic DNA extraction kit (QIAGEN and iNtRON Biotechnology). The genomic DNAs were deposited in the Plant DNA Bank in Korea (PDBK).

**Table 1:** Voucher information and summarized results of Illumina sequencing. All voucher specimens are deposited in Korea University herbarium (KUS) and DNAs are deposited in Plant DNA Bank of Korea(PDBK).

Species Name	*Voucher and DNA No.	Origin	No. of total reads	No. of plastome reads (%)	Coverage	SRA accession No.	Genbank accession No.
*Lindera aggregate* (Sims) Kosterm.	KUS & PDBK TC2016-0766	China	11,103,960	254,591 (2.29%)	460x	SRR10278376	MG581437
*Lindera angustifolia* Cheng	KUS and PDBK TC2016-0750	China	10,660,534	500,489 (4.69%)	896x	SRR10278375	MG581438
*Lindera chunii* Merr.	KUS and PDBK TC2016-0600	China	11,293,148	175,535 (1.55%)	316x	SRR10278374	MG581439
*Lindera communis* Hemsl.	KUS and PDBK TC2016-0705	China	7,962,440	238,891 (3.00%)	444x	SRR10278373	MG581440
*Lindera erythrocarpa* Makino	KUS and PDBK 2009–0331	Korea	13,056,868	254,993 (1.95%)	463x	SRR10278390	MG581441
*Lindera floribunda* (C.K.Allen) H.P.Tsui	KUS and PDBK TC2016-0843	China	10,080,482	132,581 (1.32%)	251x	SRR10278372	MG581442
*Lindera glauca* Blume	KUS and PDBK 2008–0161	Korea	13,233,342	472,332 (3.57%)	859x	SRR10278389	MG581443
*Lindera megaphylla* Hemsl.	KUS and PDBK TC2016-0760	China	8,538,694	237,461 (2.78%)	448x	SRR10278371	MG581444
*Lindera metcalfiana* C.K.Allen	KUS and PDBK TC2016-0637	China	10,364,924	24,271 (0.23%)	44x	SRR10278388	MG581445
*Lindera nacusua* (D.Don) Merr.	KUS and PDBK TC2016-0755	China	10,127,414	202,038 (1.99%)	383x	SRR10278387	MG581446
*Lindera neesiana* Kurz	KUS and PDBK TC2016-0992	China	10,013,000	70,298 (0.70%)	130x	SRR10278386	MG581447
*Lindera obtusiloba* Blume	KUS and PDBK 2008–0249	Korea	11,099,286	719,703 (6.48%)	1,303x	SRR10278378	MG581448
*Lindera praecox* (Siebold & Zucc.) Blume	KUS and PDBK TJ2016-0569	Japan	10,196,164	226,020 (2.22%)	428x	SRR10278382	MG581449
*Lindera pulcherrima* (Nees) Hook.f. var. *attenuata* Allen	KUS and PDBK TC2016-0706	China	9,292,806	302,013 (3.25%)	553x	SRR10278385	MG581450
*Lindera reflexa* Hemsl.	KUS and PDBK TC2016-0796	China	11,435,296	160,995 (1.41%)	290x	SRR10278384	MG581451
*Lindera rubronervia* Gamble	KUS and PDBK TC2016-1008	China	9,373,718	44,297 (0.47%)	82x	SRR10278383	MG581452
*Lindera sericea* Blume	KUS and PDBK 2016–0087	Korea	11,873,432	1,113,518 (9.38%)	2,020x	SRR10278377	MG581453
*Actinodaphne lancifolia* (Blume) Meisn.	KUS and PDBK 2008–0063	Korea	9,318,814	532,526 (5.71%)	1,005x	SRR10278381	MG581436
*Litsea japonica* (Thunb.) Juss.	KUS and PDBK 2012–0606	Korea	9,786,900	286,821 (2.93%)	542x	SRR10278380	MG581454
*Sassafras tzumu* (Hemsl.) Hemsl.	KUS and PDBK TC2016-0759	China	7,740,482	110,698 (1.43%)	206x	SRR10278379	MG581455

### Sequencing and annotation

Approximately 100 ng of extracted DNA were used for library construction and raw sequence reads were generated using Illumina MiSeq using reagent kit v3 (600-cycles) (Illumina, Inc. San Diego, CA). The raw read sequence data was deposited on NCBI Sequence Read Archive (SRA) under acc. nos. SRR10278371 –SRR10278390 (Table 1). The numbers of paired-end-reads of 20 new complete plastome sequences ranged from 7,740,482 in *Sassafras tzumu* to 13,233,342 in *Lindera glauca* (Siebold & Zucc.) Blume (Table 1). The average read length after trimming ranged from 258 to 287bp depending on the samples. For trimming and normalization of raw reads, BBDuk version 37.64 and BBNorm version 37.64, which were adopted in Geneious v. 11.1.2 (Biomatters Ltd.)**[52]** were used with kmer length of 27. All repeated reads were removed from trimmed reads by normalization process. The normalized reads are subjected to de novo assembly and then plastome contigs were recovered. All repeated reads were mapped to the plastome contigs and finally a single plastome contig was recovered for *L*. *obtusiloba*. The complete plastome of *L*. *obtusiloba* was assembled de novo at first. For other 19 plastomes, only the plastid reads were collected from trimmed reads using *L*. *obtusiloba* as a reference. The collected plastid reads were subjected to de novo assembly. In this way, a single plastome contig, which covers the whole plastome, was generated for the other 19 species. Annotation and mapping of protein coding genes (including exons and introns) and rRNA genes were performed using a BLAST search in the National Center for Biotechnology Information (NCBI). All tRNA genes were annotated using the tRNAscan-SE program **[53]**. Pseudogenes and deletions were determined by NCBI BLAST. The circular plastome maps were constructed by OrganellarGenomeDraw (OGDRAW)**[54]**.

### Plastome analysis

To locate the SIs of the 20 sequenced plastomes, we first identified the palindromic repeats that form the stem with longer than 4 bp loop regions using REPuter **[55]**. Their secondary structures and free energy were estimated using the mFOLD program **[56]**. For the same stem region, we identified it as SI if different species showed distinct loop sequence orientations. All of the 20 whole plastome sequences were aligned using MAFFT v. 7.017 **[57]**.

Sliding window analysis was conducted to generate the nucleotide diversity (Pi) of complete Laureae genomes using DnaSP v. 6.10 software **[58]**. The step-size was set to 200 bp, with a 600 bp window length. All of the whole plastome sequences were aligned using MAFFT v. 7.017 **[57]**. The simple sequence repeats (SSRs) were detected with the Phobos v. 3.3.12 program **[59]**. We counted the SSR if it is repeated more than ten times for mono-, five times for di-, four times for tri-, three times for tetra-, and two times for penta-SSR loci.

### Phylogenetic analysis

For the phylogenetic analysis of Lauraceae, we selected and downloaded 29 complete plastome sequences (28 Lauraceae and one Calycanthaceae plastomes) from the NCBI database (S1 Table). Out of the 29 complete plastome sequences, 11 are indicated as unverified sequences in the NCBI database. These sequences were used only for the construction of the Lauraceae phylogenetic tree using 49 taxa. The phylogenetic analysis was performed on a data set that includes 77 protein-coding genes and four rRNA genes. The 81 gene sequences were aligned separately with MUSCLE in Geneious v. 11.1.2 (Biomatters Ltd.)**[52]** and then concatenated as a single data matrix. We also constructed a phylogenetic tree for the 33 core Lauraceae group using 33 whole plastome sequences including all noncoding regions. The whole plastome sequences including all noncoding regions were aligned as a single data matrix with MAFFT v. 7.017 in Geneious v. 11.1.2. The GTR base substitution model was adopted based on the jModelTest2 **[60]** for maximum likelihood (ML) tree reconstruction using RAxML v. 7.7.1 **[61]**.

## Results and discussion

### Structures of the *Lindera* plastome

The ratio of plastid reads/total reads are ranged from 0.23% in *L*. *metcalfiana* C.K.Allen to 9.38% in *L*. *sericea* (Siebold & Zucc.) Blume (Table 1). The differences are primary due to the leaf developmental stages. The sequencing coverage of 20 complete plastomes ranged from 44x (*L*. *metcalfiana*) to 2,020x (*L*. *sericea*) (Table 1). We recovered single plastome contig, which covers whole plastome, on de novo assembly even for the lowest covered *L*. *metcalfiana*. It is primarily due to the long lead lengths and high coverage depths of Illumina MiSeq sequencing in our study.

**Fig 1:**
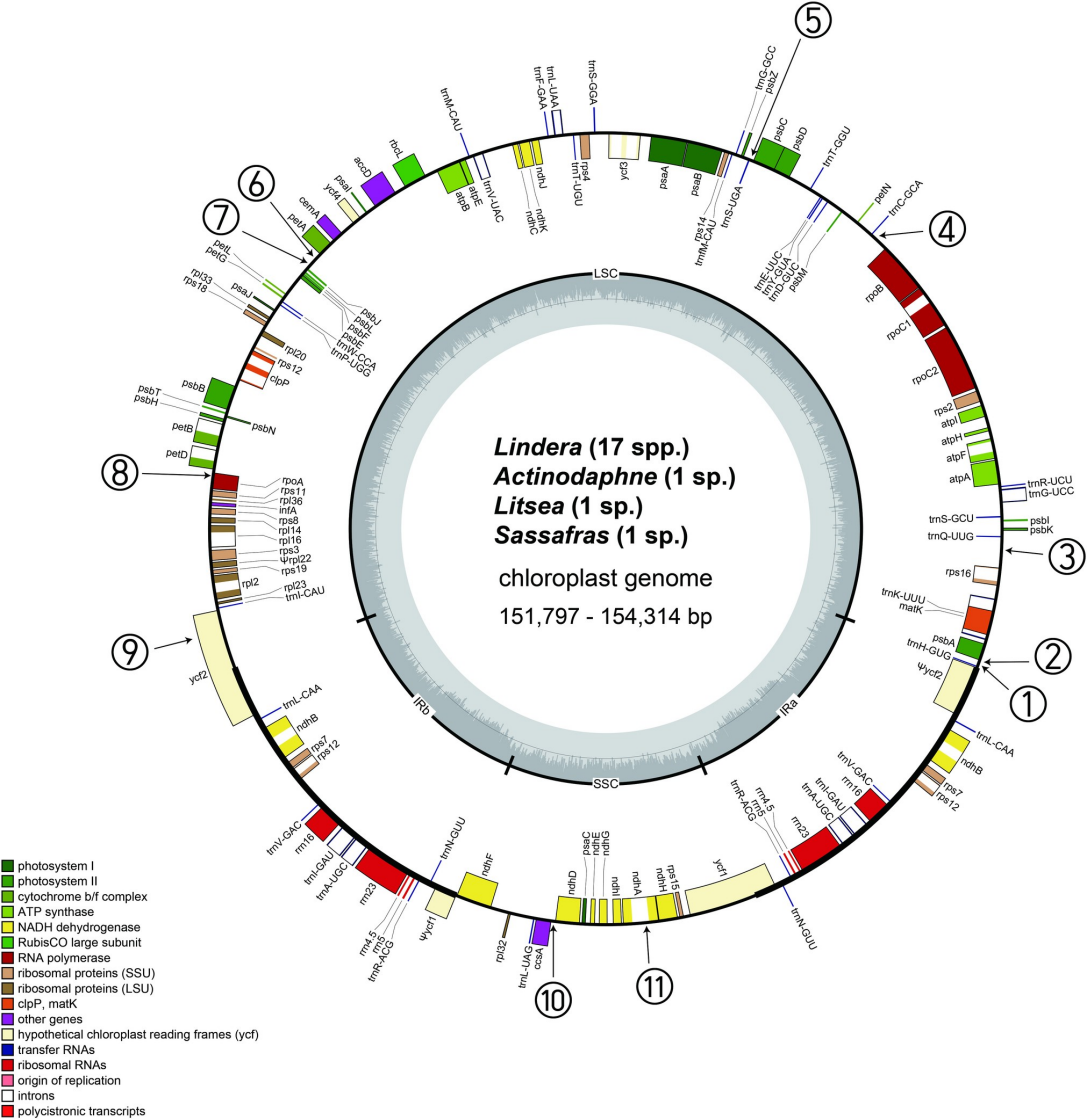
Circular plastome maps of 17 *Lindera* and three related specices. The length of the *L*. *neesiana* plastome (152,502 bp) was the shortest, the length of *Sassafras tzumu* plastome (151,797 bp) was the longest. The gene contents of 20 plastomes of *Lindera* and related genera are similar to general angiosperm plastome with two minor modifications. First, all 20 plastomes have pseudogenized *rpl*22 gene instead of true gene. Second, *rpl*23 gene was also pseudogenized in *L*. *megaphylla* and *L*. *metcalfiana*. The numbers at the outermost circle indicate the locations of the 11 SIs as shown on **Fig 2**.

The gene order and structure of the 20 plastomes are similar to those of a typical angiosperm (Fig 1) **[51, 62, 63]**. The *Lindera* plastomes ranged from 152,502 bp (*L*. *neesiana* (Wall. ex Nees) Kurz) to 154,314 bp (*L*. *erythrocarpa* Makino) in length (Fig 1 and Table 2). All *Lindera* plastomes (excluding *L*. *megaphylla* Hemsl. and *L*. *metcalfiana*) comprised of 111 unique genes (77 protein-coding genes, 30 tRNA genes, and four rRNA genes). Sixteen genes had one intron and two genes (*clp*P and *ycf*3) had two introns. Seven protein-coding, seven tRNA, and four rRNA genes were duplicated in the IR regions. The A-T content of the *Lindera* plastomes was approximately 60.8% (Table 2).

**Table 2:** Summary of 17 *Lindera* and the other Lauraceae species complete plastomes.

Species Name	Total Length	LSC Region	IR Region	SSC Region	AT contents
*Lindera aggregata*	152,664	93,728	20,066	18,804	60.8%
*Lindera angustifolia*	152,832	93,726	20,082	18,942	60.8%
*Lindera chunii*	152,694	93,724	20,066	18,838	60.8%
*Lindera communis*	152,668	93,680	20,047	18,894	60.8%
*Lindera erythrocarpa*	154,314	95,302	20,071	18,870	60.9%
*Lindera floribunda*	152,551	93,532	20,107	18,805	60.8%
*Lindera glauca*	152,863	94,213	19,854	18,942	60.8%
*Lindera megaphylla*	152,762	93,676	20,065	18,956	60.8%
*Lindera metcalfiana*	152,893	93,881	20,078	18,856	60.8%
*Lindera nacusua*	152,762	93,740	20,065	18,893	60.8%
*Lindera neesiana*	152,502	93,558	20,066	18,812	60.8%
*Lindera obtusiloba*	152,772	93,702	20,080	18,910	60.9%
*Lindera praecox*	152,728	93,701	20,066	18,895	60.8%
*Lindera pulcherrima* var. *attenuata*	153,679	93,743	20,066	19,787	60.9%
*Lindera reflexa*	153,006	93,145	20,474	18,913	60.8%
*Lindera rubronervia*	152,885	93,754	20,091	18,949	60.9%
*Lindera sericea*	153,028	93,166	20,474	18,914	60.8%
*Actinodaphne lancifolia*	152,728	93,793	20,066	18,803	60.9%
*Litsea japonica*	152,718	93,696	20,066	18,890	60.9%
*Sassafras tzumu*	151,797	92,751	20,096	18,854	60.8%

Two genes were pseudogenized in *Lindera* and related genera. For example, the *rpl*22 gene is a pseudogene in all species. The *rpl*23 gene is a pseudogene in *L*. *megaphylla* and *L*. *metcalfiana*. The pseudogenized *rpl*23 was also reported in the parasitic *Cassytha* and non-parasitic *Nectandra* Rol. ex Rottb. of Lauraceae **[20, 21]**.

The contraction/expansion boundaries between SC and IR regions vary among the angiosperm species **[64]**. This causes differences in angiosperm plastome sizes. Our 20 plastomes show similar SC and IR boundaries. Therefore, the variations in length among the 20 plastomes are minor. The LSC/IR boundary is located within the *ycf*2 coding region, and the SSC/IR boundary is located within the *ycf*1 coding region, respectively. These results are consistent with previous studies for the Laureae tribe **[20, 21, 65]**.

### Small inversions in *Lindera* plastomes

**Fig 2:**
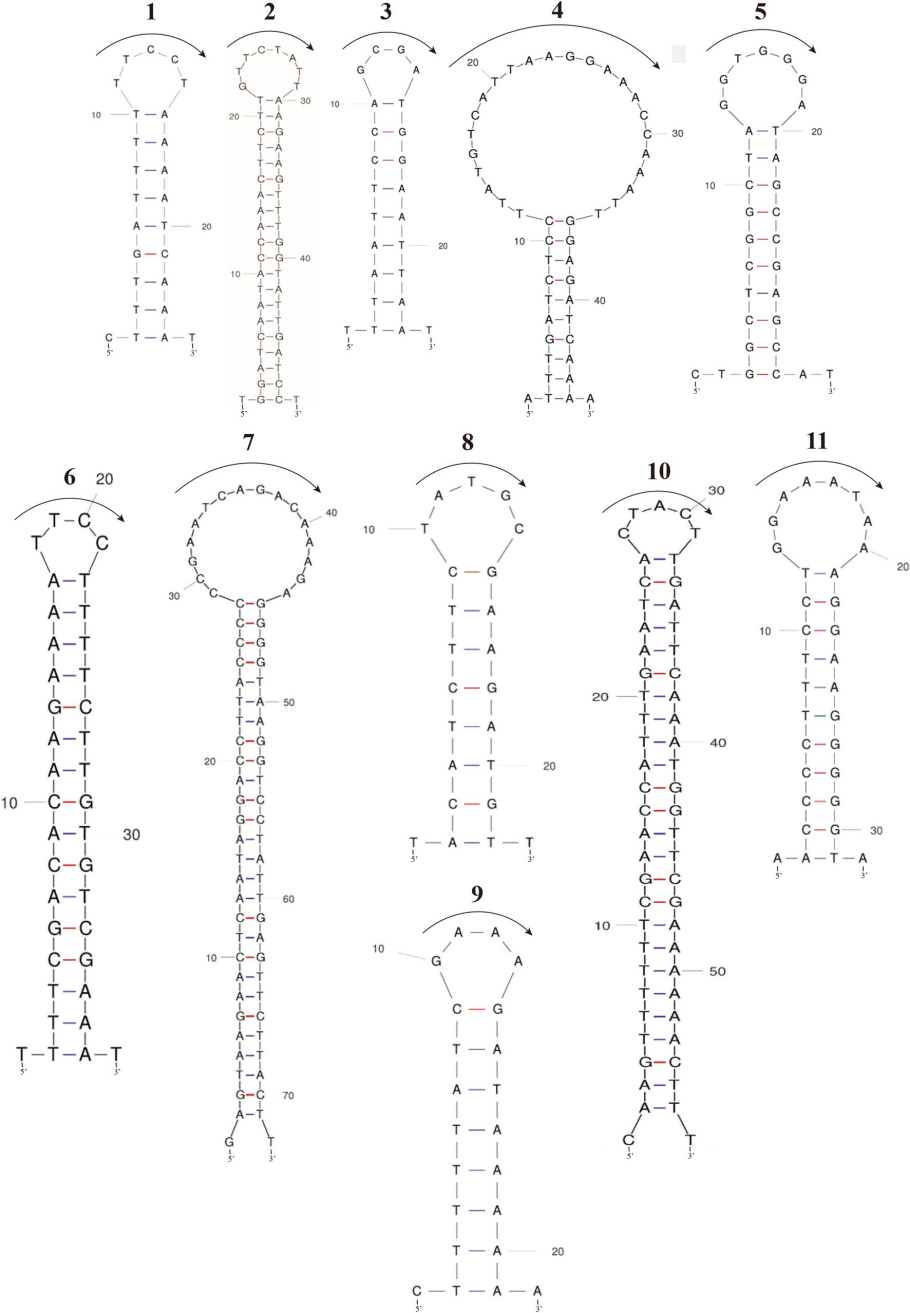
Predicted stem and loop structures of 11 small inversions. The direction of arrow above each loop indicates a common type (A form) and the counterdirection indicates rare type (B form). The locations of 11 SIs are as follows; 1 and 2 on *trn*H-*psb*A, 3 on *rps*16-*trn*Q-UUG, 4 on *rpo*B-*trn*C-GCA, 5 on *psb*C-*trn*S-UGA, 6 and 7 on *pet*A-*psb*J, 8 on *rpo*A, 9 on *ycf*2, 10 on *ccs*A-*ndh*D and 11 on *ndh*A intron. The corresponding locations of 11 SIs are also marked on **Fig 1** and their free energy values are given in Table 3.

**Fig 3:**
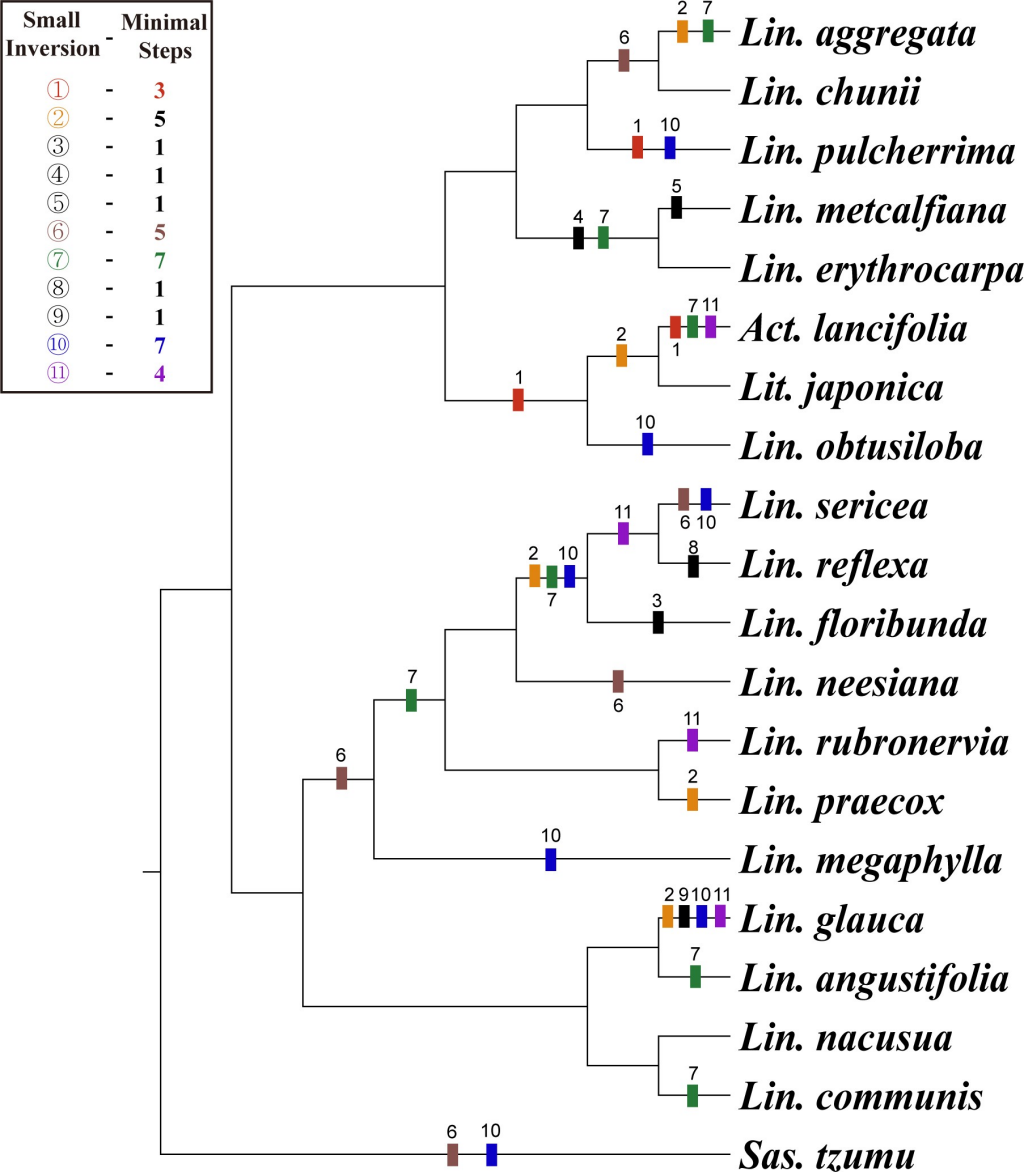
The evolutionary changes of 11 small inversions. The changes of each small inverion was plotted by ACCTRAN character transformation criteria of parsimony analysis on the Maximum likelihood (ML) tree for 20 Lauraceae based on whole plastome sequences. The aligned sequence was 156,569 bp in length. The ML tree was determined by RAxML with -ln L = 259398.416970. The number above colored bar indicates the change from A to B form and the number below colored bar indicates the changes from B to A form. The A form configurations of 11 SIs are given in Fig 2 and the B form structures are opposite direction to the arrow on the loop of A form. Detailed A and B form sequences and distribution among species are given in Table 3.

SIs are identifiable among closely related species with similar base sequences. A total of 11 SIs was identified in the 20 plastomes (Figs 2 and 3 and Table 3). Among them, eight were distributed in the IGS (*trn*H*-psb*A 1 and 2, *rps*16*-trn*Q, *rpo*B*-trn*C, *psb*C*-trn*S, *pet*A*-psb*J 1 and 2 and *ccs*A*-ndh*D), two in the gene coding region (*rpo*A and *ycf*2), and one in the intron region (*ndh*A intron). The length of the loops ranged from 4 to 24 bp. Among them, the SIs present in *trn*H-*psb*A 2, *rps*16-*trn*Q, *pet*A-*psb*J 2, *ccs*A-*ndh*D, and *ndh*A introns have been reported in *Persea* Mill., *Machilus* Nees and *Phoebe* Nees species **[14, 15, 19]**. However, six other SIs are newly reported in this study and are located in regions such as *trn*H-*psb*A 1, *rpo*B-*trn*C, *psb*C-*trn*S, *pet*A-*psb*J 1, *rpo*A and *ycf*2.

**Table 3:** Summary of 11 small inversions in 20 newly sequenced plastomes in Lauraceae.

No.	Region	Stem - Free energy value	Loop type and Sequence	Species Name
**1**	*trn*H-*psb*A1	TTTGATTTT (9bp) dG = -5.60	A_1_: TTCCT (5bp)	*Act*. *lancifolia*, *Lin*. *aggregata*, *Lin*. *angustifolia*, *Lin*. *communis*, *Lin*. *erythrocarpa*, *Lin*. *glauca*, *Lin*. *megaphylla*, *Lin*. *nacusua*, *Lin*. *praecox*, *Lin*. *rubronervia*, *Sas*. tzumu*
			A_2_: TTCAA (5bp)	*Lin*. *chunii*, *Lin*. *floribunda*, *Lin*. *metcalfiana*, *Lin*. neesiana*, *Lin*. *reflexa*, *Lin*. sericea*
			B: AGGAA (5bp)	*Lin*. *obtusiloba*, *Lin*. *pulcherrima var*. *attenuata*, *Lit*. *japonica*
**2**	*trn*H-*psb*A2	GGATCAATACCAAACTTCTT (20bp) dG = -20.59	A_1_: AATAGAAC (8bp)	*Lin*. *chunii*, *Lin*. *erythrocarpa*, *Lin*. *megaphylla*, *Lin*. *metcalfiana*, *Lin*. *neesiana*, *Lin*. obtusiloba*, *Sas*. tzumu*
			A_2_: AATAAAAC (8bp)	*Lin*. *rubronervia*
			A_3_: ATAGAA (6bp)	*Lin*. angustifolia*, *Lin*. *pulcherrima var*. attenuata*
			A_4_: ATAGAACAGAA (11bp)	*Lin*. communis*, *Lin*. nacusua*
			B_1_: GTTCTATT (8bp)	*Act*. lancifolia*, *Lin*. aggregate*, *Lin*. *floribunda*, *Lin*. *praecox*, *Lin*. *reflexa*, *Lin*. *sericea*, *Lit*. *japonica*
			B_2_: TTCTAT (6bp)	*Lin*. glauca*
**3**	*rps*16-*trn*Q	TTAATTCCA (9bp) dG = -7.30	A: GCGA (4bp)	*Act*. *lancifolia*, *Lin*. *aggregata*, *Lin*. *angustifolia*, *Lin*. *chunii*, *Lin*. *communis*, *Lin*. *erythrocarpa*, *Lin*. *glauca*, *Lin*. *megaphylla*, *Lin*. *metcalfiana*, *Lin*. *nacusua*, *Lin*. *neesiana*, *Lin*. *obtusiloba*, *Lin*. *praecox*, *Lin*. *pulcherrima var*. *attenuata*, *Lin*. *reflexa*, *Lin*. *rubronervia*, *Lin*. *sericea*, *Lit*. *japonica*, *Sas*. *tzumu*
			B: TCGC(4bp)	*Lin*. *floribunda*
**4**	*rpo*B-*trn*C-GCA	TTTGATCTCC (10bp) dG = -7.22	A_1_: TTATGTCATTAAGG AAACCAAATT (24bp)	*Act*. *lancifolia*, *Lin*. *aggregata*, *Lin*. *angustifolia*, *Lin*. *chunii*, *Lin*. *floribunda*, *Lin*. *glauca*, *Lin*. *megaphylla*, *Lin*. *neesiana*, *Lin*. *obtusiloba*, *Lin*. *praecox*, *Lin*. *pulcherrima var*. *attenuata*, *Lin*. *reflexa*, *Lin*. *rubronervia*, *Lin*. *sericea*, *Lit*. *japonica*, *Sas*. *tzumu*
			A_2_: TTATGTCATTAAGG AAACCAAATT (24bp)	*Lin*. *communis*, *Lin*. *nacusua*
			A_3_: TTATGTCATTAAGG AAACAAAATT (24bp)	*Lin*. *erythrocarpa*
			B: AATTTGGTTTCCTTA ATGACATAA (24bp)	*Lin*. *metcalfiana*
**5**	*psb*C-*trn*S-UGA	TGGCTCGGCTA (11bp) dG = -12.50	A: GGTGGGA (7bp)	*Act*. *lancifolia*, *Lin*. *aggregata*, *Lin*. *angustifolia*, *Lin*. *chunii*, *Lin*. *communis*, *Lin*. *erythrocarpa*, *Lin*. *floribunda*, *Lin*. *glauca*, *Lin*. *megaphylla*, *Lin*. *nacusua*, *Lin*. *neesiana*, *Lin*. *obtusiloba*, *Lin*. *praecox*, *Lin*. *pulcherrima var*. *attenuata*, *Lin*. *reflexa*, *Lin*. *rubronervia*, *Lin*. *sericea*, *Lit*. *japonica*, *Sas*. *tzumu*
			B: TCCCACC (7bp)	*Lin*. *metcalfiana*
**6**	*pet*A-*psb*J	TTTCGACACAAGAAAA (16 bp) dG = -15.98	A: TTCC (4bp)	*Act*. *lancifolia*, *Lin*. angustifolia*, *Lin*. *communis*, *Lin*. *erythrocarpa*, *Lin*. *glauca*, *Lin*. *metcalfiana*, *Lin*. *nacusua*, *Lin*. *neesiana*, *Lin*. *obtusiloba*, *Lin*. *pulcherrima var*. *attenuata*, *Lin*. *sericea*, *Lit*. *japonica*
			B_1_: GGAA (4bp)	*Lin*. *aggregata*, *Lin*. *chunii*, *Lin*. *floribunda*, *Lin*. *megaphylla*, *Lin*. *praecox*, *Lin*. *reflexa*, *Lin*. *rubronervia*
			B_2_: GCGGAAAATT (10bp)	*Sas*. *tzumu*
**7**	*pet*A-*psb*J	AGTAAGAACTCAATAGGACCTTACCCCT (28bp) dG = -30.19	A: CTTTGTCTGATTCG (14bp)	*Lin*. *chunii*, *Lin*. *floribunda*, *Lin*. *glauca*, *Lin*. *megaphylla*, *Lin*. *nacusua*, *Lin*. *obtusiloba*, *Lin*. *pulcherrima var*. *attenuata*, *Lin*. *reflexa*, *Lin*. *sericea*, *Lit*. *japonica*, *Sas*. *tzumu*
			B -CCGAATCAGACAAAGA (16bp)	*Act*. *lancifolia*, *Lin*. *aggregata*, *Lin*. *angustifolia*, *Lin*. *communis*, *Lin*. *erythrocarpa*, *Lin*. *metcalfiana*, *Lin*. *neesiana*, *Lin*. *praecox*, *Lin*. *rubronervia*
**8**	*rpo*A	ACATCTTC (8bp) dG = -6.90	A: TATGC (5bp)	*Act*. *lancifolia*, *Lin*. *aggregata*, *Lin*. *angustifolia*, *Lin*. *chunii*, *Lin*. *communis*, *Lin*. *erythrocarpa*, *Lin*. *floribunda*, *Lin*. *glauca*, *Lin*. *megaphylla*, *Lin*. *metcalfiana*, *Lin*. *nacusua*, *Lin*. *neesiana*, *Lin*. *obtusiloba*, *Lin*. *praecox*, *Lin*. *pulcherrima var*. *attenuata*, *Lin*. *rubronervia*, *Lin*. *sericea*, *Lit*. *japonica*
			B: GCATA (5bp)	*Lin*. *reflexa*
**9**	*ycf*2	TTTTTATC (8bp) dG = -5.45	A: GAAA (4bp)	*Act*. *lancifolia*, *Lin*. *aggregata*, *Lin*. *angustifolia*, *Lin*. *chunii*, *Lin*. *communis*, *Lin*. *erythrocarpa*, *Lin*. *floribunda*, *Lin*. *megaphylla*, *Lin*. *metcalfiana*, *Lin*. *nacusua*, *Lin*. *neesiana*, *Lin*. *obtusiloba*, *Lin*. *praecox*, *Lin*. *pulcherrima var*. *attenuata*, *Lin*. *reflexa*, *Lin*. *rubronervia*, *Lin*. *sericea*, *Lit*. *japonica*, *Sas*. *tzumu*
			B: TTTC (4bp)	*Lin*. *glauca*
**10**	*ccs*A-*ndh*D	AAGTTTTTTCGAACCATTTGAATCA (25bp) dG = -27.14	A: CTACT (5bp)	*Act*. *lancifolia*, *Lin*. *aggregata*, *Lin*. *angustifolia*, *Lin*. *chunii*, *Lin*. *communis*, *Lin*. *erythrocarpa*, *Lin*. *metcalfiana*, *Lin*. *nacusua*, *Lin*. *neesiana*, *Lin*. *praecox*, *Lin*. *rubronervia*, *Lin*. *sericea*, *Lit*. *japonica*
			B: AGTAG (5bp)	*Lin*. *floribunda*, *Lin*. *glauca*, *Lin*. *megaphylla*, *Lin*. *obtusiloba*, *Lin*. *pulcherrima var*. *attenuata*, *Lin*. *reflexa*, *Sas*. *tzumu*
**11**	*ndh*A intron	ACCCCTTTCCT (11bp) dG = -9.23	A_1_: GGAAATAA (8bp)	*Lin*. *aggregata*, *Lin*. *angustifolia*, *Lin*. *chunii*, *Lin*. *communis*, *Lin*. *erythrocarpa*, *Lin*. *floribunda*, *Lin*. *megaphylla*, *Lin*. *metcalfiana*, *Lin*. *nacusua*, *Lin*. *neesiana*, *Lin*. *obtusiloba*, *Lin*. *praecox*, *Lin*. *pulcherrima var*. *attenuata*, *Lit*. *japonica*
			A_2_: GGAAAGAA (8bp)	*Sas*. *tzumu*
			B_1_: TTATTTCC (8bp)	*Act*. *lancifolia*, *Lin*. *glauca*, *Lin*. *reflexa*, *Lin*. *sericea*
			B_2_: TTATTTAC (8bp)	*Lin*. *rubronervia*

The asterisk (*) indicates species with modified stem sequence.

SIs are not easily found because they are usually located in the spacer regions and do not affect the gene order. Therefore, most plastome research do not mentioned the SIs. The presence of SIs was reported in the *trn*H-*psb*A, *pet*A-*psb*J and *trn*L-F regions **[44, 45, 48, 50]**. Not only in these regions, but the SIs occur extensively in angiosperm plastomes **[31, 40]**. Kim and Lee (2005) compared the complete plastomes of four species of Poaceae and reported 16 SI regions **[31]**. Thereafter, SIs have been reported in studies of Araliaceae **[49]**, Arecaceae **[50]**, Lauraceae **[14, 15, 18, 19]**, Lamiaceae **[51]**, and Oleaceae **[37]**. Using the complete plastome sequences of 12 genera and 29 species, Dong et al. (2012) proposed 23 regions with high divergence as regions where SIs may exist **[66]**. Our six of eleven SIs were located in the five suggested regions (*trn*H-*psb*A, *trn*Q-*rps*16, *rpo*B-*trn*C, *pet*A-*psb*J and *ndh*A intron), but five SIs are located in other regions. The majority (nine of 11) of *Lindera* SIs were located on downstream of genes. The other two SIs are located gene coding region (no. 9) and intron region (no. 11). Six of 11 SIs were located downstream of two adjacent genes where the 3′ ends of the two genes met tail-to-tail. However, the stem forming regions of SIs were closer to the 3′ end of one of the genes. The other three SIs (nos. 1–3 in Fig 1) were located in the intergenic spacers between genes that had the same orientation (tail-to head orientation). These locations are generally accorded the previous prediction of SI locations in other plants **[36]**. The main function of stem-loop forming SI is the stability maintenance of the transcribed mRNA **[36]**.

We also estimated the free energy (-ΔG) of each SI regions using the MFOLD program (Table 3). Two different orientations of all 11 SIs show identical free energy values. As a result, the flip-plop mutations of SIs are selectively neutral in evolution. Therefore, the flip-plop mutations occur easily on the same locus. We also estimated the number of flip-plop mutations for each SI on the ML tree using the ACCTRAN criteria of parsimony analysis (Fig 3). Five (nos. 3, 4, 5, 8, and 9) out of 11 SIs are autapomorphic characters. The other six SIs are synapomorphic characters, but their character states are changed several times ranging from five to seven times (Fig 3). As expected by free-energy values, the multiple changes of each SI character explain by easy flip-plop mutation at the stem region of hairpin structure. Therefore, the SIs are not strong phylogenetic markers to define the monophyletic groups. But, it shows strong molecular identification powers at species level.

SIs are always bounded with the hairpin structure of DNA sequences. The flip-plop mutation at the stem region create different orientation of loop sequences **[63]**. A single flip-plop mutation at the stem region generate several base pair differences at the loop region. Therefore, care should be used when regions where SIs are included, as these are used in the construction of phylogenetic trees, because incorrect phylogenetic signals may be given by such regions **[63]**. In particular, some areas, such as *trn*H-*psb*A **[44, 45]**, which are often used as interspecies markers, might be better to use after the removing the SI(s).

### Plastome divergence hotspot regions

**Fig 4:**
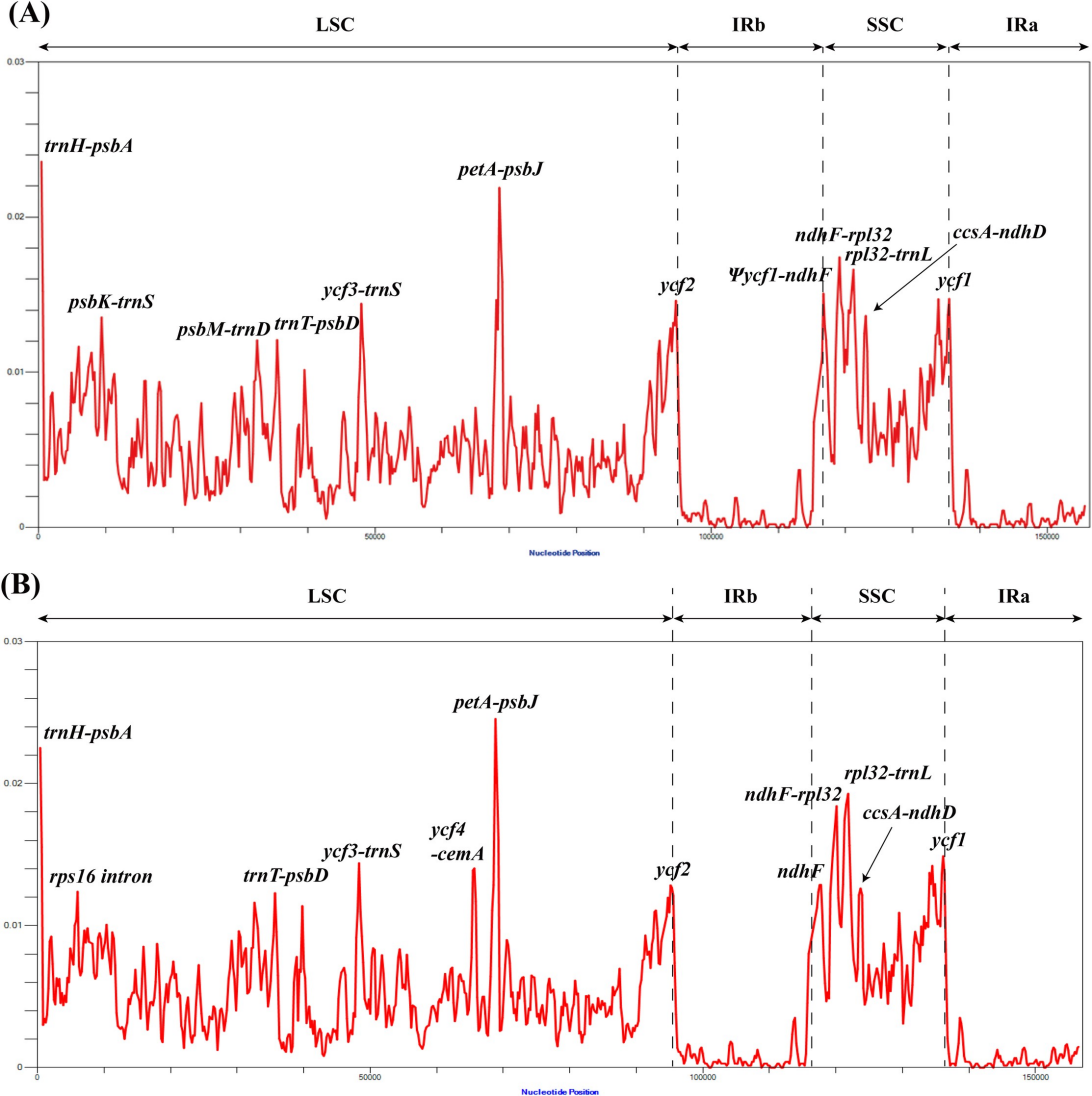
Comparison of high Pi value regions by sliding window analysis. (A) Sliding window analysis showing high Pi value regions among the complete plastomes of 17 *Lindera* species. (B) Sliding window analysis showing high Pi value regions among the complete plastomes of 24 Laureae species. The window length was 600 bp and the step size was 200 bp in both analyses. The X-axis represents the midpoint position of each window, while the Y-axis represents nucleotide divergence (Pi) values in each window.

To evaluate the level of nucleotide divergence of *Lindera* and other Laureae members, nucleotide diversities (Pi) among 17 *Lindera* (Fig 4A) and 24 Laureae complete plastomes (Fig 4B and S1 Table) were calculated with DnaSP v 6.10 software **[58]**. Among the 17 *Lindera* plastomes, Pi values ranged from 0 to 0.02358 (*trn*H*-psb*A). The highest Pi value of gene and intron regions was recorded on *ycf*1 (0.01473) and on the *rps*16 intron (0.01165), respectively. Five regions show Pi values higher than 0.015 and these regions were located in the IGS region (Fig 4A). These regions were *trn*H-*psb*A (0.02358), *pet*A-*psb*J (0.02189), *ndh*F-*rpl*32 (0.01741), *rpl*32-*trn*L (0.01662) and Ψ*ycf*1-*ndh*F (0.01507). The zero Pi values on a 600 bp sliding window were recorded in nine sites of the IR region.

Among 24 Laureae plastomes (S1 Table), Pi values ranged from 0 to 0.02455 (*pet*A-*psb*J) (Fig 4B). The highest Pi value of gene and intron regions was recorded on *ycf*1 (0.01489) and on the *rps*16 intron (0.01239), respectively. Four regions showed Pi values higher than 0.015 and all of these regions were located in the IGS region (Fig 4B). These regions were *pet*A-*psb*J (0.02455), *trn*H-*psb*A (0.02252), *rpl*32-*trn*L (0.01929) and *ndh*F-*rpl*32 (0.01841). The zero Pi values on a 600 bp sliding window were recorded in three sites of the IR region.

Both analyses clearly show that the IR regions are more conserved compared to LSC and SSC regions. The results are consistent with previous reports from diverse angiosperms **[15, 17–19, 49, 51, 67]**. Yi and Kim (2012) indicated this as a positional effect **[51]**. This is thought to be attributable to frequent recombination between two copies in the IR region to continuously remove mutations. In plastid genomes, this positional effect acts more strongly than functional factors such as gene coding sequences (CDS), IGS, and intron regions. However, the functional effects act more strongly in the same LSC and SSC regions. For instance, all the regions with high Pi values mentioned above correspond to the IGS regions, not the CDS region, and most of the regions with Pi values exceeding 0.01 not mentioned above are also located in the IGS region (Fig 4). In the CDS region, Pi values were shown to be relatively high in the *ndh*F, *ycf*2 and *ycf*1 regions located at the LSC-IR-SSC junction, and this is considered attributable to IR contraction/expansion.

In order to test the usefulness of the high Pi value regions for phylogenetic and DNA barcoding studies, we constructed the phylogenetic tree using the combined sequences of *pet*A-*psb*J, *trn*H-*psb*A, *ndh*F-*rpl*32 and *rpl*32-*trn*L-UAG intergenic spacer regions (IGS). Each of the four IGS regions shows Pi values more than 0.18. The aligned sequence of four regions was 4,734 bp in length and a ML (-ln *L =* 12859.707713) tree with bootstrap values more than 50% internal nodes were presented in S1 Fig. The tree shows almost fully resolved topology even the bootstrap supporting values are low on some internal nodes. Therefore, using the regions with high Pi values presented in this study will be helpful for studies of genealogy between closely related species or DNA barcoding studies to distinguish species.

### Types and distribution of simple sequence repeats

**Fig 5:**
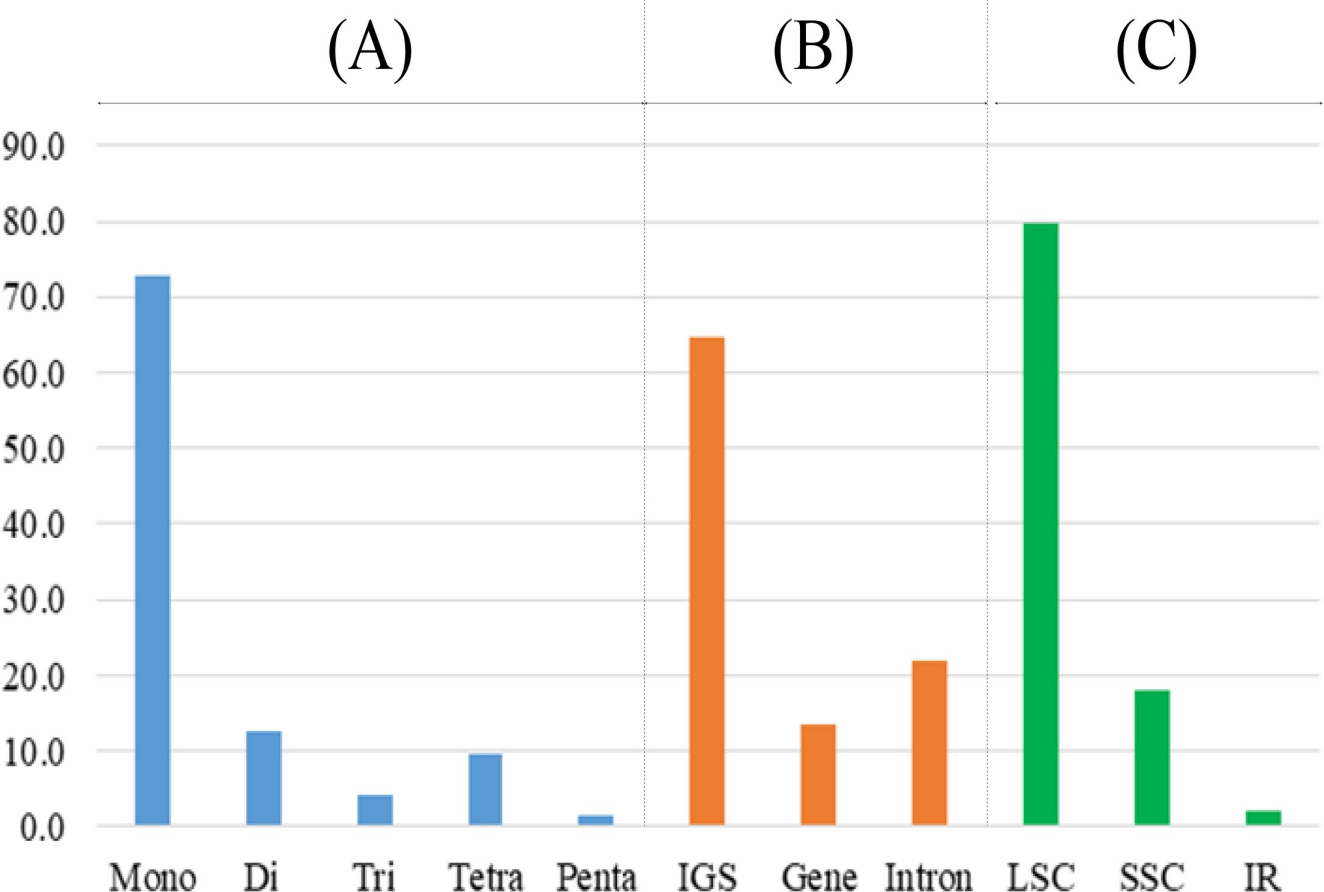
Types and distribution of simple sequence repeats (SSRs) for 17 *Lindera* and three related species. (A) Distribution patterns of SSR types in 20 complete plastomes. (B) and (C) Average distribution patterns of SSRs on the partitioned regions of plastome. The X-axis indicates SSR types or partitioned plastome regions, while Y-axis indicates the numbers of SSRs.

Plastid simple sequence repeats (SSRs) have been used for molecular markers in plant population genetic studies **[51, 68, 69]** because they show high intraspecific variations. The copy number differences of SSRs are usually due to the slippage-mispairing during DNA replication **[70]**. In this study, we analyzed the SSRs of 20 newly sequenced plastomes (Fig 5 and S2 Table). An average of 83 SSRs were detected in each plastome. The numbers ranged from 73 in *L*. *nacusua* (D.Don) Merr. to 91 in *L*. *angustifolia* (W.C.Cheng) Nakai (S2 Table). The majority of SSRs were mono-SSRs and accounted for 72.7% of total SSRs. The di-SSRs comprised 12.4%, followed by tetra- (9.4%), tri- (4.2%), and penta-SSRs (1.3%) (Fig 5). The length of mono-SSRs ranged from 10 to 31 bp. Also, 31 A bases were detected in the *ycf*3 intron 1 of the *L*. *metcalfiana* plastome. The average number of mono-SSRs was 60.2, with the largest number being 65 in *L*. *erythrocarpa*, *L*. *neesiana* and *L*. *pulcherrima* (Nees) Hook.f. var. *attenuate* C.K.Allen, and the smallest number being 47 in *L*. *nacusua* (S2 Table). The length of di-SSRs ranged from 10 to 20 bp, and (AT)n was the most common type of di-SSR (S2 Table). The tetra-SSRs occurred on an average of 7.8 sites of each plastome and were more common than the tri-SSRs. This result is consistent with the previous results of complete plastomes of Lauraceae **[15, 17]**.

SSRs were scattered along the Laureae plastomes (Fig 5 and S2 Table). Of these SSRs, 64.6% were located in IGS regions and 21.8% occurred in intron regions. In contrast, only 13.6% were located in CDS regions (Fig 5). These results were similar to other studies of Lauraceae **[18]**. We also partitioned the distribution of SSRs according to the LSC, SSC and IR regions, and found that 79.8% of the SSRs were located in the LSC region (61.3%) (Fig 5). Only one or two SSRs were located in the IR region (S2 Table). SSRs were completely absent from the IR regions of *L*. *communis* Hemsl., *L*. *nacusua* and *L*. *pulcherrima* var. *attenuata* (S2 Table).

In order to evaluate the phylogenetic utility of SSRs, we compare the locus of each di-, tri-, tetra-, and penta-SSRs among 20 species (S3 Table). Eight of 44 loci were conserved among all species. Twenty-two loci show autapomorphic status and 14 loci show synapomorphic status. We also plotted each synapomorpic locus on the phylogenetic tree and only two (nos. 16 and 41, S3 Table) of them support monophyly of a clade consisted of *L*. *glauca*, *L*. *angustifolia*, *L*. *nacusua*, and *L*. *communis*. Other 12 synapomorphic loci are changed multiple times ranged from two to 10 times (Tree not shown). Therefore, the phylogenetic utilities of the SSR loci are very low in *Lindera*. But, it is a good maker to the identification of species. Actually, we were confidently identified all 20 species using the 44 SSR locus.

### Phylogenetic analysis

**Fig 6:**
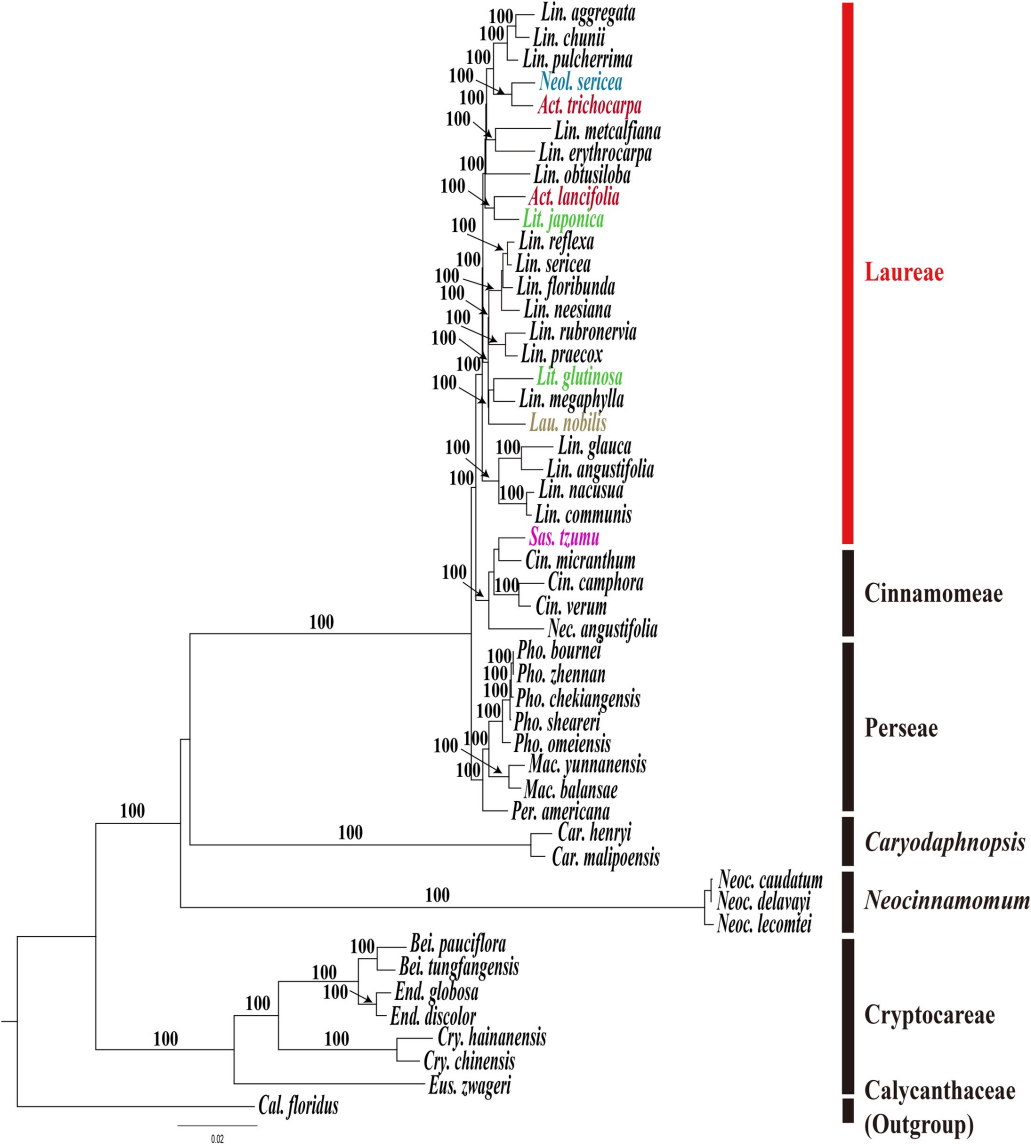
Maximum likelihood (ML) tree for 49 Lauraceae based on 77 protein-coding and four rRNA gene sequences of plastome. The aligned sequence was 73,193 bp in length. The ML tree was determined by RAxML with -ln *L =* 294926.225755. The number on each node indicates the ML bootstrap value above 95% supports. Abbreviations: *Act*. *= Actinodaphne*, *Bei*. *= Beilschmidedia*, *Cal*. *= Calycanthus*, *Car*. *= Caryodaphnopsis*, *Cin*. *= Cinnamomum*, *Cry*. *= Cryptocarya*, *End*. *= Endiandra*, *Eus*. *= Eusideroxylon*, *Lau*. *= Laurus*, *Lin*. *= Lindera*, *Lit*. *= Litsea*, *Mac*. *= Machilus*, *Nec*. *= Nectandra*, *Neoc*. *= Neocinnamomum*, *Neol*. *= Neolitsea*, *Per*. *= Persea*, *Pho*. *= Phoebe* and *Sas*. *= Sassafras*.

To validate the phylogenetic relationships of Lauraceae, we aligned gene coding sequences for 49 Lauraceae taxa (S1 Table). The concatenated 81 gene sequences were 73,386 bp in length. The ML tree was obtained by RAxML with -ln *L =* 294926.225755. Most internal nodes are supported by 100% ML bootstrap values (Fig 6). Phylogenetic analysis was also performed on a data set that included whole plastome sequences for the core 33 Laureae taxa. The aligned whole plastome sequence including all noncoding regions was 158,484 bp in length. The 33 core Laureae tree was also constructed using the same condition as above (S2 Fig). In addition, we also construct the phylogenetic tree using only the intergenic spacer (IGS) regions of plastomes for 33 core Lauraceae. The aligened IGS sequence was 46,162 bp in length. The ML tree (-ln *L =* 92207.119754) was determined by same method as above (S3 Fig). The whole plastome tree (S2 Fig) and the IGS tree (S3 Fig) are identical for the phylogenetic relationships among 33 core Lauraceae. The tree topologies base on three different data set (Fig 6 and S2 and S3 Figs) also show almost identical relationships and only difference was the different levels of bootstrp support values at some internal nodes.

The trees suggested that the tribe Laureae was a monophyletic group, and that it is a sister group to the tribe Cinnamomeae. Their close outgroup is the tribe Perseae. In contrast to monophyletic tribes, 17 *Lindera* species formed paraphyletic assemblages because they include members of other genera such as *Laurus (Lau*.*)*, *Litsea (Lit*.*)*, *Neolitsea (Neol*.*)*, and *Actinodaphne (Act*.*)* (Fig 6 and S2 Fig). For example, *Act*. *tricocarpa* C.K.Allen forms a sister group with *Neol*. *Sericea* (Blume) Koidz., while *Act*. *lancifolia* forms a sister group with *Lit*. *japonica*. Furthermore, *Lit*. *glutinosa* (Lour.) C.B.Rob. forms a clade with *L*. *megaphylla* Hemsl. and *Lau*. *nobilis* L. Neither *Actinodaphne* nor *Litsea* species form a monophyletic group in our tree (Fig 6 and S2 Fig).

The genus *Sassafras* is usually treated as a member of the tribe Laureae based on general morphology, but it nested in a clade with *Cinnamomum* Schaeff. *Nectandra* was sister genus to the paraphyletic *Cinnamomum* in our plastome trees (Fig 6 and S2 Fig). Previous phylogenetic studies using partial plastid gene sequences **[6, 27–29]** or complete protein coding gene sequences **[20]** also reported the same relationships as in our tree. Therefore, our plastome trees agreed that *Sassafras* should be included in the tribe Cinnamomeae rather than the tribe Laureae **[20]**.

Previous phylogenetic studies of Laureae using different molecular markers also support the paraphyly of *Lindera*. For examples, plastid *mat*K tree showed the *Lindera* genus do not form a monophyletic group because some species of *Litsea* and *Actinodaphne* nested within a large *Lindera* clade **[26]**. The ITS and ETS tree also showed the strong paraphyly of *Lindera* because *Lindera* species were occurred at least four different clades **[27]**. Two of the clades also includes some species of *Litsea*, *Actinodaphne*, *Parasassafras*, *Sinosassafras*, and *Iteadaphne*. In addition, the nuclear *rpb*2 tree also show strong paraphyletic natures of *Lindera* **[71]**. Our whole plastome data also supports not only the paraphyly of *Lindera* but the paraphyly of other genera of Laureae. Therefore, the generic boundaries of tribe Laureae defined by morphological characters should be revised in near future.

Most of the Lauraceae are evergreen trees or shrubs and are distributed in tropical and subtropical regions of East Asia **[3, 8–10]**. Deciduousness was reported for some temperate *Lindera* and *Litsea* species and three *Sassafras* species **[3, 8–10]**. In order to test the evolution of deciduousness in *Lindera* and related genera, we plotted the character status on the whole plastome tree (S2 Fig). The tree clearly indicated that deciduousness was emerged at least five times and one reverse evolution from deciduous to evergreen also occurred on a branch leading to *L*. *floribunda* (C.K.Allen) H.P.Tsui. The deciduous trees *L*. *obtusiloba* Blume and *L*. *erythrocarpa* are derived independently from different evergreen ancestors. A core deciduous clade including six *Lindera* species from *L*. *sericea* to *L*. *praecox* (Siebold & Zucc.) Blume also include an evergreen, *L*. *floribunda*. Furthermore, some of these species also show semi-deciduous status depending on the distribution range **[3, 9, 10]**. Therefore, distribution range expansion to the north or high elevation and distribution range contraction to the south or lower elevation is the primary driving force of leaf characteristics in the evolution of *Lindera*. In addition, global climate change such as ice ages and global warming are also responsible for the evolution of leaf characteristics.

To test whether the problem of tangled relationships in which the genera in the Laureae are mixed with each other caused by SIs, a phylogenetic tree was constructed under the same tree building options, excluding the 11 SI regions that were found in this study (S4 Fig). However, there was no effect on the phylogenetic outcome. Similar results can be seen in the analysis using only the gene coding regions containing nine *Lindera* species instead of the whole plastome sequences used in this study **[23]**. Therefore, these results along with the previous phylogenetic study of *Lindera* and related genera probably suggest that hybridizations and plastome captures occurred frequently in the process of differentiation of these genera and species. In order to confirm the degree of plastome capture, additional studies including suitable nuclear markers are needed for *Lindera*, *Litsea*, *Actinodaphne*, and *Laurus*. Alternatively, the plastome phylogeny may indicate that the morphological characters defined the genus originated for several times.

## Supporting information

S1 Fig Maximum likelihood (ML) tree based on four intergenic spacer (IGS) region with the more than 0.18 PI values. The four IGS regions are *trn*H-*psb*A, *pet*A-*psb*J, *ndh*F-*rpl*32 and *rpl*32-*trn*L-UAG and the aligned sequence was 4,734 bp in length. The ML tree among 33 core Lauraceae was determined by RAxML progran with -ln *L =* 12859.707713. The number on each node indicates the ML bootstrap values with more than 50% support. Abbreviations: *Act*. *= Actinodaphne*, *Cin*. *= Cinnamomum*, *Lau*. *= Laurus*, *Lin*. *= Lindera*, *Lit*. *= Litsea*, *Mac*. *= Machilus*, *Per*. *= Persea*, *Pho*. *= Phoebe* and *Sas*. *= Sassafras*.
(TIF)

S2 Fig Maximum likelihood (ML) tree for 33 core Lauraceae based on whole plastome sequences. The aligned sequence was 157,779 bp in length. The ML tree was determined by RAxML with -ln *L =* 283016.993074. The number on each node indicates the ML bootstrap value above 95% supports. Orange colored node and branch indicate the evolution of deciduous leaf habits (D), while black colored node and branch indicate evergreen leaf habits (E). Abbreviations: *Act*. *= Actinodaphne*, *Cin*. *= Cinnamomum*, *Lau*. *= Laurus*, *Lin*. *= Lindera*, *Lit*. *= Litsea*, *Mac*. *= Machilus*, *Per*. *= Persea*, *Pho*. *= Phoebe* and *Sas*. *= Sassafras*.
(TIF)

S3 Fig Maximum likelihood (ML) tree based on the combined intergenic spacer (IGS) region of 33 core Lauraceae. The aligened sequence was 46,162 bp in length. The ML tree was determined by RAxML with -ln *L =* 92207.119754. The number on each node indicates the ML bootstrap values with more than 70% support. Abbreviations: *Act*. *= Actinodaphne*, *Cin*. *= Cinnamomum*, *Lau*. *= Laurus*, *Lin*. *= Lindera*, *Lit*. *= Litsea*, *Mac*. *= Machilus*, *Per*. *= Persea*, *Pho*. *= Phoebe* and *Sas*. *= Sassafras*.
(TIF)

S4 Fig Maximum likelihood (ML) tree based on whole plastome sequences excluding 11 samll inversion sites of 33 core Lauraceae. The aligned sewuence was 143,135 bp in length. The ML tree was determined by RAxML with -ln *L =* 251467.252384. The number on each node indicates the ML bootstrap values with more than 80% support. Abbreviations: *Act*. *= Actinodaphne*, *Cin*. *= Cinnamomum*, *Lau*. *= Laurus*, *Lin*. *= Lindera*, *Lit*. *= Litsea*, *Mac*. *= Machilus*, *Per*. *= Persea*, *Pho*. *= Phoebe* and *Sas*. *= Sassafras*.
(TIF)

S1 Table The list of 49 plastome sequences used in this study. The NCBI accession numbers with bold face are newly reported from this study and the numbers with normal face are reported from previous studies by other authors and downloaded from NCBI database for this study. The accession number with an asterisk indicates unverified sequences, marked by NCBI database. The unverified sequences are newly annotated by current authors before used in this study.
(XLSX)

S2 Table The numbers of various SSR types and SSR distribution along the plastome of 20 Laureae taxa. (XLSX)

S3 Table The distribution patterns of simple sequence repeat (SSR)s among 20 Lindera and related genera. *Note indicate conserved (con.), autapomorphic (aut.) and synapomorphic (syn) characters.
(XLSX)
