# Supplementary figures and images for: Characterization of 20 complete plastomes from the tribe Laureae (Lauraceae) and distribution of small inversions
Source: PLoS One. 2019 Nov 1;14(11):e0224622. doi: 10.1371/journal.pone.0224622 (PMC6824564; doi:10.1371/journal.pone.0224622)

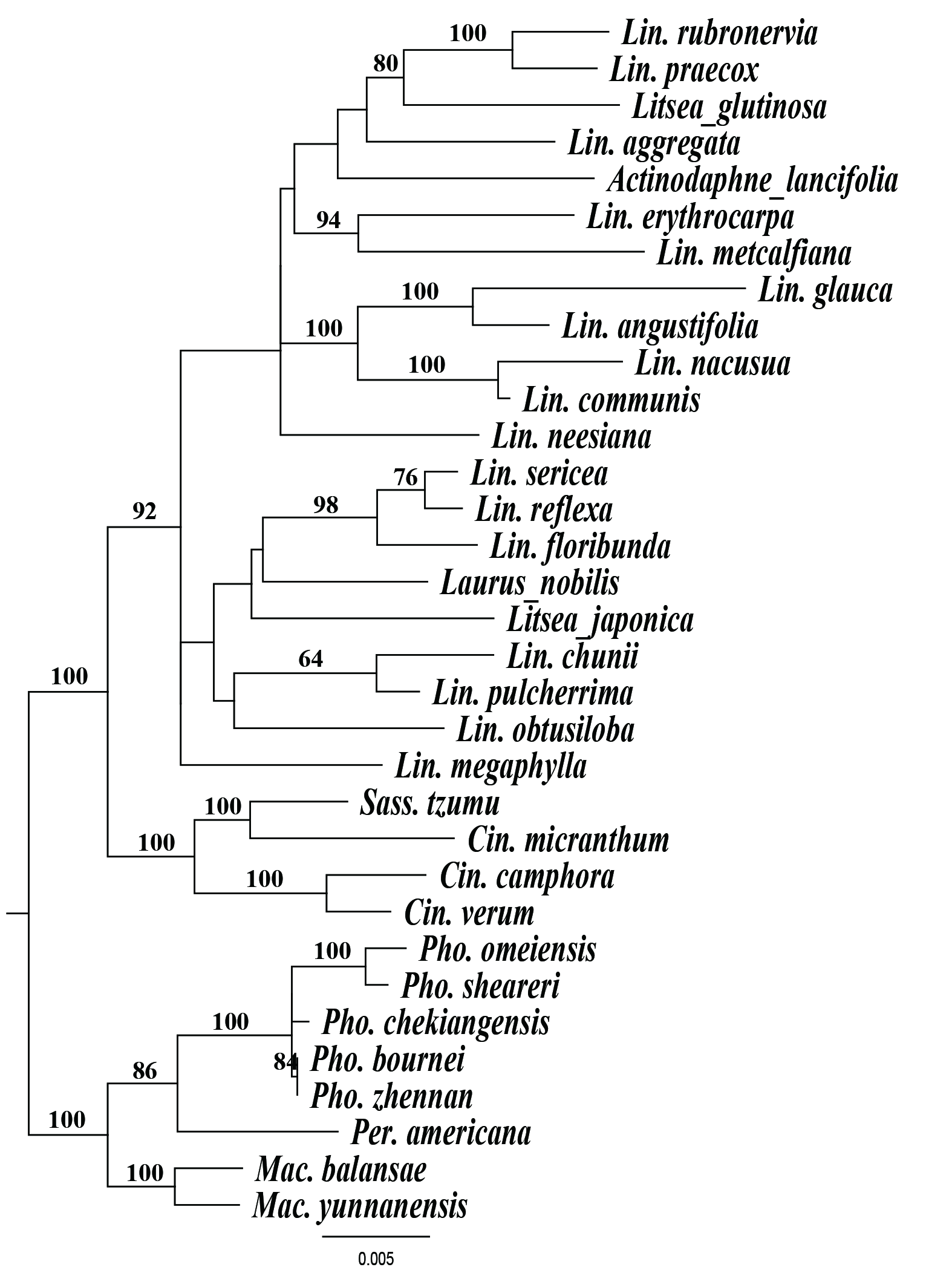

Supplement: S1 Fig — The four IGS regions are trnH-psbA, petA-psbJ, ndhF-rpl32 and rpl32-trnL-UAG and the aligned sequence was 4,734 bp in length. The ML tree among 33 core Lauraceae was determined by RAxML progran with -ln L = 12859.707713. The number on each node indicates the ML bootstrap values with more than 50% support. Abbreviations: Act. = Actinodaphne, Cin. = Cinnamomum, Lau. = Laurus, Lin. = Lindera, Lit. = Litsea, Mac. = Machilus, Per. = Persea, Pho. = Phoebe and Sas. = Sassafras. (TIF) [file pone.0224622.s001.tif]

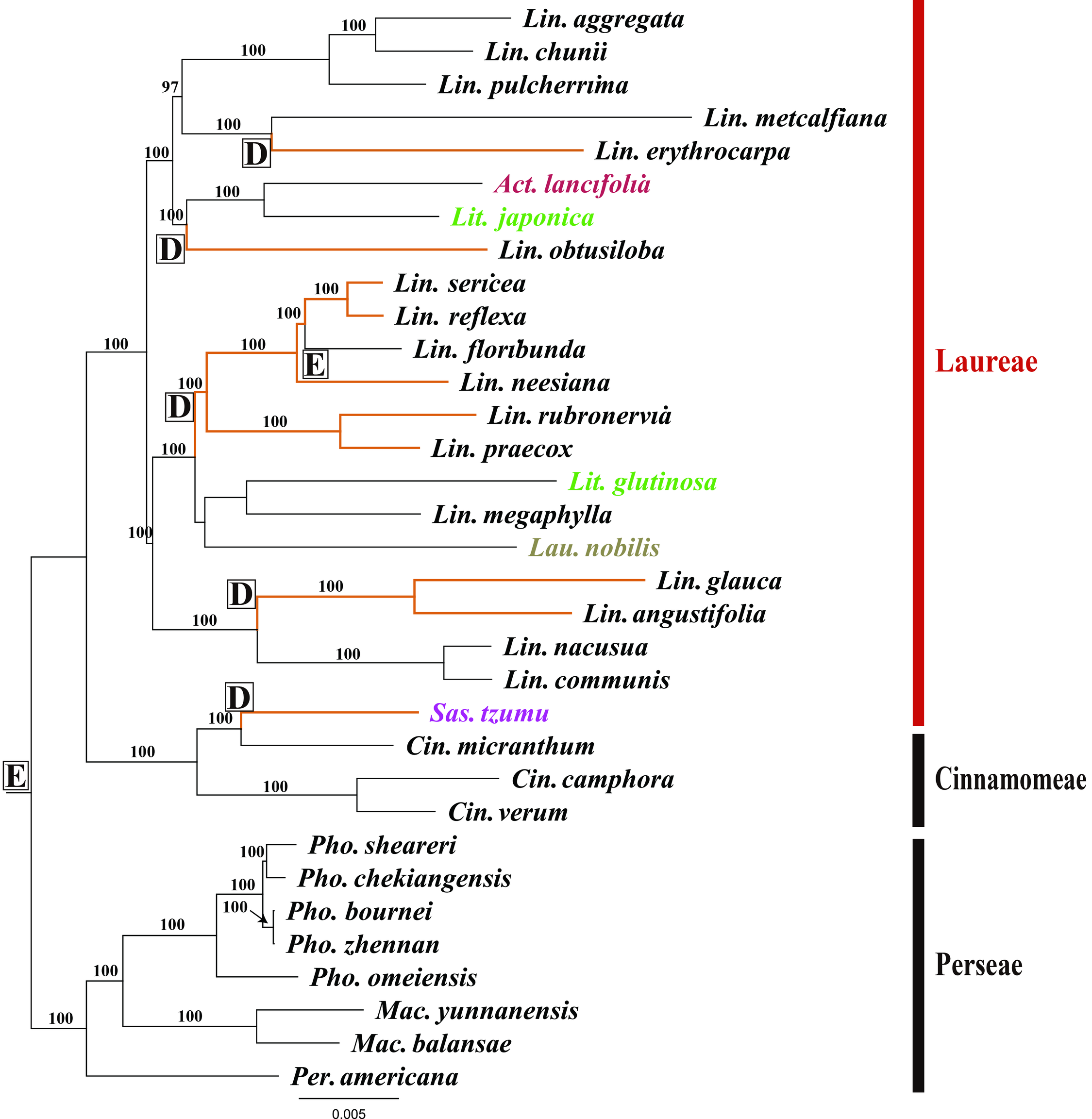

Supplement: S2 Fig — The aligned sequence was 157,779 bp in length. The ML tree was determined by RAxML with -ln L = 283016.993074. The number on each node indicates the ML bootstrap value above 95% supports. Orange colored node and branch indicate the evolution of deciduous leaf habits (D), while black colored node and branch indicate evergreen leaf habits (E). Abbreviations: Act. = Actinodaphne, Cin. = Cinnamomum, Lau. = Laurus, Lin. = Lindera, Lit. = Litsea, Mac. = Machilus, Per. = Persea, Pho. = Phoebe and Sas. = Sassafras. (TIF) [file pone.0224622.s002.tif]

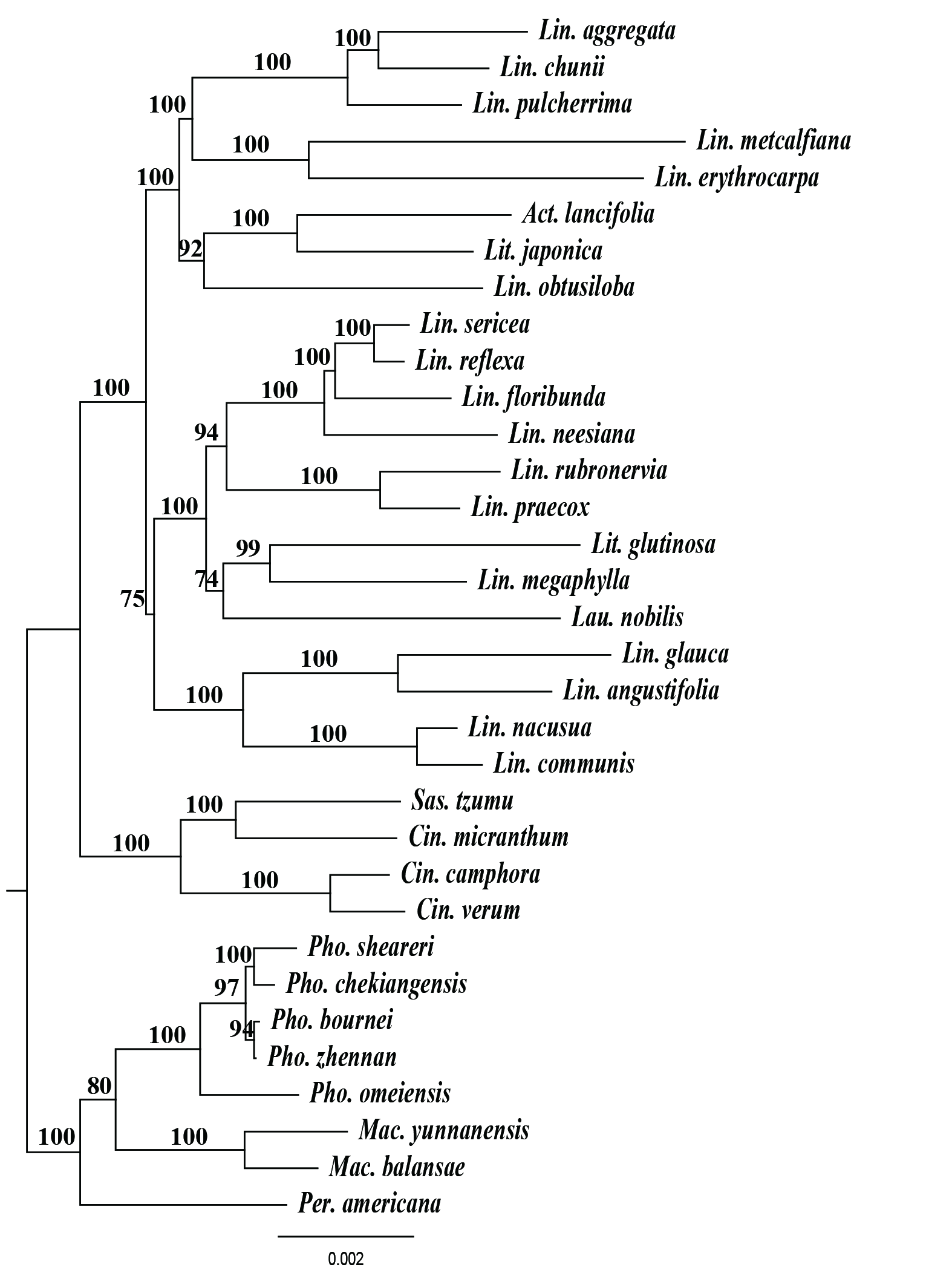

Supplement: S3 Fig — The aligened sequence was 46,162 bp in length. The ML tree was determined by RAxML with -ln L = 92207.119754. The number on each node indicates the ML bootstrap values with more than 70% support. Abbreviations: Act. = Actinodaphne, Cin. = Cinnamomum, Lau. = Laurus, Lin. = Lindera, Lit. = Litsea, Mac. = Machilus, Per. = Persea, Pho. = Phoebe and Sas. = Sassafras. (TIF) [file pone.0224622.s003.tif]

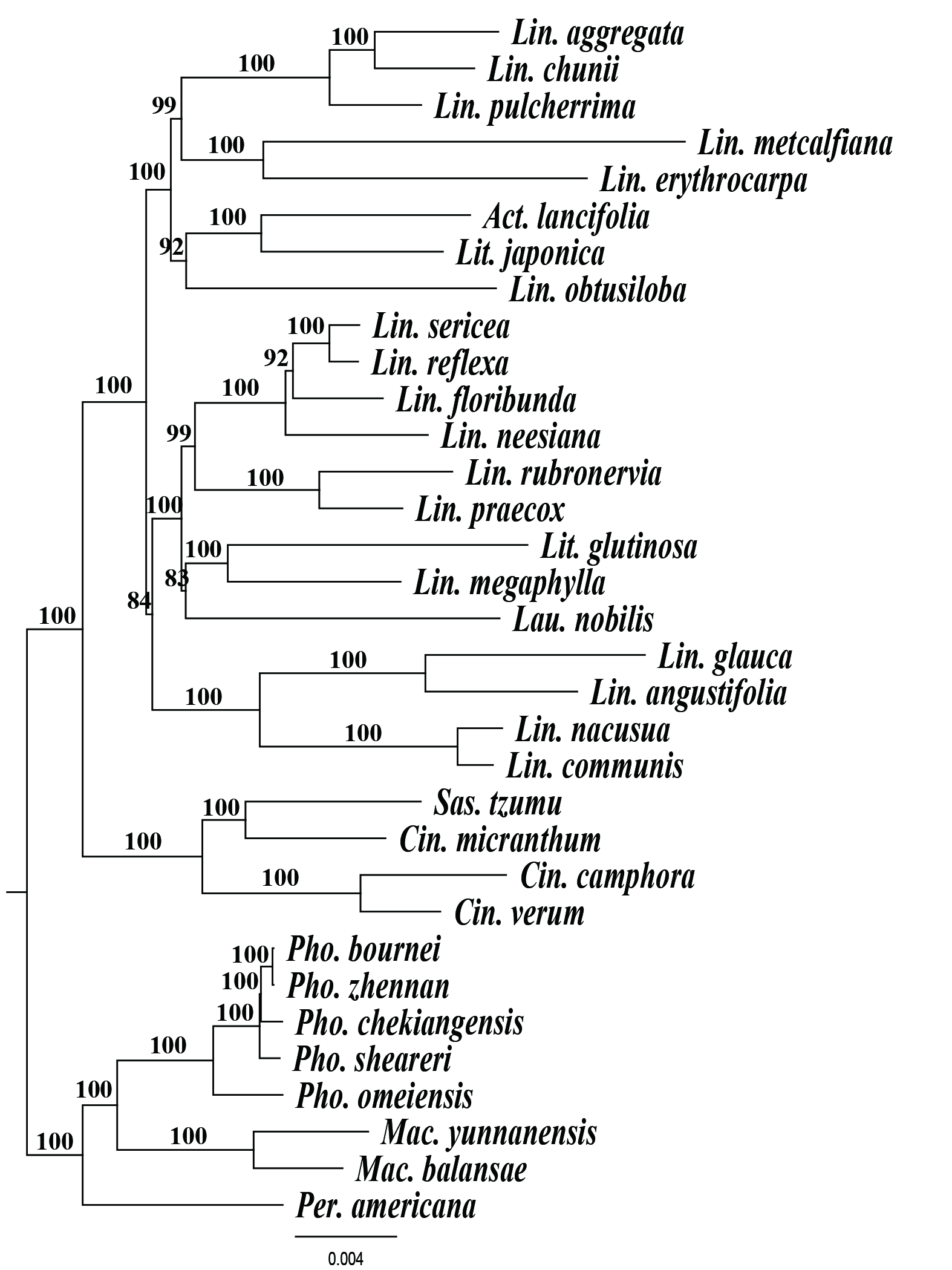

Supplement: S4 Fig — The aligned sewuence was 143,135 bp in length. The ML tree was determined by RAxML with -ln L = 251467.252384. The number on each node indicates the ML bootstrap values with more than 80% support. Abbreviations: Act. = Actinodaphne, Cin. = Cinnamomum, Lau. = Laurus, Lin. = Lindera, Lit. = Litsea, Mac. = Machilus, Per. = Persea, Pho. = Phoebe and Sas. = Sassafras. (TIF) [file pone.0224622.s004.tif]
